# *QuickStats*: Percentage* of Children and Adolescents Aged ≤17 Years Who Had Ever Received a Diagnosis of Concussion or Brain Injury,^†^ by Sex and Age Group — National Health Interview Survey,^§^ United States, 2022

**DOI:** 10.15585/mmwr.mm7233a5

**Published:** 2023-08-18

**Figure:**
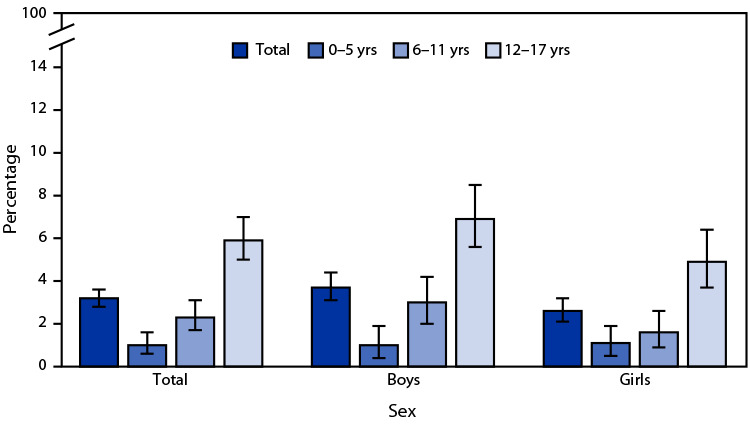
In 2022, 2.3 million (3.2%) children and adolescents aged ≤17 years had ever received a diagnosis of a concussion or brain injury. Diagnosis of a concussion or brain injury increased with age, from 1.0% among those aged 0–5 years to 2.3% among those aged 6–11 years, and 5.9% among those aged 12–17 years. Percentages were higher for boys than girls overall (3.7% versus 2.6%), among those aged 6–11 years (3.0% versus 1.6%), and those aged 12–17 years (6.9% versus 4.9%) but were similar by sex among those aged 0–5 years (1.0% versus 1.1%).

**Source:** National Center for Health Statistics, National Health Interview Survey, 2022. https://www.cdc.gov/nchs/nhis.htm

For more information on this topic, CDC recommends the following link: https://www.cdc.gov/traumaticbraininjury/

